# Sequential activation of M1 and M2 phenotypes in macrophages by Mg degradation from Ti-Mg alloy for enhanced osteogenesis

**DOI:** 10.1186/s40824-022-00262-w

**Published:** 2022-04-28

**Authors:** Luxin Liang, Deye Song, Kai Wu, Zhengxiao Ouyang, Qianli Huang, Guanghua Lei, Kun Zhou, Jian Xiao, Hong Wu

**Affiliations:** 1 State Key Laboratory of Powder Metallurgy, Central South University, Changsha, 410083 People’s Republic of China; 2 Department of Orthopedics, The Second Xiangya Hospital, Central South University, Changsha, 410011 People’s Republic of China; 3 Department of Rehabilitation, Xiangya Hospital, Central South University, Changsha, 410008 People’s Republic of China; 4 Foshan (Southern China) Institute for New Materials, Foshan, 528200 People’s Republic of China; 5 Department of Orthopedics, Xiangya Hospital, Central South University, Changsha, 410008 People’s Republic of China; 6 School of Mechanical and Aerospace Engineering, Nanyang Technological University, Singapore, 639798 Singapore; 7 Department of Pharmacy, Xiangya Hospital, Central South University, Changsha, 410008 People’s Republic of China; 8 National Clinical Research Center for Geriatric Disorders, Xiangya Hospital, Central South University, Changsha, 410008 People’s Republic of China

**Keywords:** Mg degradation, Inflammation, Macrophage, Polarization, Osteogenesis

## Abstract

**Background:**

Even though the modulatory effects of Magnisum (Mg) and its alloys on bone-healing cells have been widely investigated during the last two decades, relatively limited attention has been paid on their inflammation-modulatory properties. Understanding the activation process of macrophages in response to the dynamic degradation process of Mg as well as the relationship between macrophage phenotypes and their osteogenic potential is critical for the design and development of advanced Mg-based or Mg-incorporated biomaterials.

**Methods:**

In this work, a Ti-0.625 Mg (wt.%) alloy fabricated by mechanical alloying (MA) and subsequent spark plasma sintering (SPS) was employed as a material model to explore the inflammatory response and osteogenic performance in vitro and in vivo by taking pure Ti as the control. The data analysis was performed following Student’s t-test.

**Results:**

The results revealed that the macrophages grown on the Ti-0.625 Mg alloy underwent sequential activation of M1 and M2 phenotypes during a culture period of 5 days. The initially increased environmental pH (~ 8.03) was responsible for the activation of M1 macrophages, while accumulated Mg^2+^ within cells contributed to the lateral M2 phenotype activation. Both M1 and M2 macrophages promoted osteoblast-like SaOS-2 cell maturation. In vivo experiment further showed the better anti-inflammatory response, regenerative potentiality and thinner fibrous tissue layer for the Ti-0.625 Mg alloy than pure Ti.

**Conclusion:**

The results highlighted the roles of Mg degradation in the Ti-0.625 Mg alloy on the sequential activation of macrophage phenotypes and the importance of modulating M1-to-M2 transition in macrophage phenotypes for the design and development of inflammation-modulatory biomaterials.

**Supplementary Information:**

The online version contains supplementary material available at 10.1186/s40824-022-00262-w.

## Introduction

Bone loss caused by trauma, tumor and infection is one of the most common injuries in clinic. So far, the employment of artificial bone substitutes has been considered as a promising strategy for bone defect treatment [1]. After the implantion of a biomaterial at an injury site, the bone regeneration process involves multiple phases including coagulation, inflammation, bone formation and remodeling initiates [2, 3]. Previous works focused on the direct modulatory role of bone biomaterials on bone-forming cells such as mesenchymal stem cells (MSCs) and osteoblasts [4, 5]. However, recent investigations have highlighted the impact of the biomaterial-induced inflammatory response on subsequent osteogenesis [6, 7]. Although an appropriate acute inflammatory response is generally considered to support and/or benefit bone healing, uncontrollable and chronic inflammation is detrimental and causes delayed bone regeneration or fibrous encapsulation [8, 9]. Hence, adjusting biomaterial properties to modulate the inflammatory response for facilitating bone healing is regarded as a novel design concept for the next-generation bone biomaterials [10].

As cells of innate immunity, macrophages are among the first batch of cells recruited to biomaterial surface after the implantation surgery. The high plasticity of macrophages allows them to switch their functions from one to another under various kinds of stimuli such as biomaterial surface characteristics, bioactive inorganic ions and immuno-modulatory biomolecules [11–13]. According to the surface markers and functions, macrophages could be roughly classified as pro-inflammatory M1 and anti-inflammatory M2 phenotypes [14]. This molecular mechanism underlying inflammation-mediated osteogenesis is considered to be associated with the release behavior of macrophages in response to biomaterials [15]. In general, M1 macrophages contribute to osteogenesis through the initiation of the acute inflammatory stage [16], clearance of debris at the injury site and direct secretion of chemokines such as C-C chemokine ligand 2 (CCL2), CXC motif ligand 8 (CXCL8) and stromal cell-derived factor 1 (SDF-1) for the recruitment of bone-healing cells which include MSCs, osteoprogenitor and vascular progenitor cells [6, 17]. Comparatively, M2 macrophages benefit wound healing by secreting anti-inflammatory cytokines for inflammation resolution and growth factors such as bone morphogenetic proteins (BMPs), vascular endothelial growth factors (VEGFs) and transforming growth factor-β (TGF-β) to enhance the differentiation and function of bone-healing cells [8, 10, 18]. Recent studies have found that M1 macrophages could also directly contribute to the osteogenic differentiation of MSCs and maturation of osteoblasts via the production of oncostatin M (OSM), BMP-2 and BMP-6 [19, 20]. This indicates that the relationship between the macrophage phenotype and growth factor release remain unveiled. Thus, further exploring the pro-healing roles of M1 and M2 macrophages is critical for the development of inflammation-modulatory biomaterials with enhanced therapeutic efficacy.

Magnesium (Mg) and its alloys are potential candidate materials for orthopedic application due to their unique characteristics such as similar relative density and elastic modulus to those of human bone tissue, high degrees of biocompatibility as well as degradability which can avoid the requirement for a secondary removal surgery [21, 22]. Even though the modulatory effects of Mg and its alloys on bonehealing cells which are directly responsible for osteogenesis and vascularization have been extensively studied during the last two decades [23], relatively limited attention has been paid on their inflammation-modulatory properties [24, 25]. The degradation of Mg in physiological environment is commonly accompanied by the release of Mg^2+^, an increase in environmental pH and the production of H_2_ [26]. Differing from the anti-inflammatory role of Mg^2+^, an alkaline micro-environment (pH ~ 8.2) was found to be pro-inflammatory [27, 28]. Previous works reported that macrophages treated by micron-size Mg-2.1Nd-0.2Zn-0.5Zr particles tended to be polarized to the M1 phenotype, while the extract of Mg micro-particles of which the pH was adjusted to 7.4 induced M2 polarization in macrophages [29, 30]. These controversial results indicated that the released Mg^2+^ and OH^−^ during Mg degradation exhibited an antagonism effect on macrophage activation. Thus, it is speculated that the polarization state of macrophages cultured on the Mg surface may be continuously variable due to the dynamic degradation process of Mg. However, Mg and its alloys possess high initial corrosion rates which can lead to significant cytotoxicity, and the extract of Mg alloys is usually employed to evaluate cell compatibility for in vitro experiments [31]. Thus, it is difficult to monitor the initial activated state of macrophages grown on the Mg surface. To design and develop advanced inflammation-regulatory Mg alloys and Mg-containing bone biomaterials, it is essential to understand the dynamic relationship between macrophage polarization and Mg degradation.

In our previous work, Ti-xMg (x = 0.312, 0.625, 1.25 and 2.5 wt.%) alloys were fabricated by mechanical alloying (MA) and subsequent spark plasma sintering (SPS) [32]. The cytocompatibility of the Ti-xMg alloys was found to be adjustable by modulating the adding amount of Mg. The Ti-0.625 Mg alloy exhibited favorable biocompatibility [32]. Thus, this alloy was used as a material model to study the activation process of macrophages in response to Mg degradation by taking pure Ti as the control in this study. In addition, the relationship between macrophage polarization state and its corresponding osteo-immunomodulatory effect was evaluated.

## Materials and methods

### Fabrication and characterization of the Ti-0.625 Mg alloy

The Ti-0.625 Mg alloy was prepared as previously reported [32]. In brief, the mixed powder of Ti and Mg was milled for 8 h under the argon atmosphere. Thereafter, the as-milled powders were sintered firstly at 600 °C for 180 s and then at 800 °C for 30 s in a SPS furnace (FCT D25/3, Germany). The heating rate and pressure were set as 100 °C/min and 30 MPa, respectively. The alloy was cut into discs with a diameter of 14 mm and thickness of 1 mm for the subsequent in vitro experiment and cylinder-shaped implants with a diameter of 2 mm and length of 15 mm for the in vivo test. The surfaces of various specimens were polished by abrasive paper to 1500 # for the in vitro cell experiment.

An electron probe micro-analyzer (EPMA; JXA-8230, Japan) was employed to analyze the distribution of Mg. The in-depth microstructure of specimens prepared by mechanical grinding and subsequent ion-thinning was evaluated by a transmission electron microscope (TEM; Tecnai G^2^ F20 S-TWIN, USA) equipped with an energy dispersive x-ray (EDX) system. The surface roughness of each specimen was analyzed using atomic force microscopy (AFM, Bruker Dimension Icon, USA).

### Analysis of Mg degradation in the Ti-0.625 Mg alloy

Each Ti-0.625 Mg disc was immersed in 1 mL Dulbecco’s modified eagle medium (DMEM; USA) and kept in a cell incubator operated under the condition of 5% CO_2_, 95% humidity and 37 °C. The culture medium was exchanged every day, and the supernatants of day 1, 3 and 5 were collected for Mg^2+^ concentration and pH value measurements. The concentration of Mg^2+^ was measured using inductively coupled plasma atomic emission spectroscopy (ICP-AES; Spectro blue sop, Germany). The pH value was evaluated using a pH meter (OHAUS, China).

### Cell culture

Murine-derived RAW 264.7 macrophages obtained from China Infrastructure of Cell Line Sources were cultured in DMEM supplemented with 10% fetal bovine serum (FBS; Gibco USA) and 1% penicillin/streptomycin (PS; Gibco USA). Human osteoblastic SaOS-2 cells (China Infrastructure of Cell Line Sources) were incubated in McCoy’s medium (Gibco, USA) containing 15% FBS and 1% PS. Both types of cells were incubated under a standard condition (5% CO_2_, 95% humidity and 37 °C).

### Macrophage response to the Ti-0.625 Mg alloy

To assess macrophage response to the Ti-0.625 Mg alloy, each specimen was placed in 24-well plate and seeded with RAW 264.7 cells at a density of 60,000 cells per well. At pre-defined time points, the culture medium for macrophages was collected and filtered through 220 nm filter membranes. Afterwards, the conditioned medium (CM) was obtained by mixing above culture medium with fresh McCoy’s complete medium at a volume ratio of 1:6 [20].

#### Cell morphology and proliferation

The morphology of RAW 264.7 cells cultured on various specimen surfaces was observed using scanning electron microscopy (SEM; FEI Quanta FEG250, Japan). After being cultured for 1, 3 and 5 days, the cells were successively washed by phosphate buffer solution (PBS), fixed with 2.5% glutaraldehyde (Sigma, USA), dehydrated in ethanol solutions with gradient concentrations (from 30 to 100%, 10 min at each concentration) and treated by graded tertiary butanol solutions (25, 50, 75 and 100%, 10 min at each concentration). After subsequent freeze-drying and gold-sputtering, the cells were observed by SEM.

The proliferation of RAW 264.7 cells cultured on various specimens was evaluated by living cell staining. After being cultured for 1, 3 and 5 days, the cells were stained in 2 μM calcein-AM (Dojindo, Japan) solution at 37 °C for 15 min. Thereafter, the stained cells were imaged under a fluorescence microscope (Leica, Germany). Quantitative results were obtained from the fluorescence images using Image-Pro Plus 6.0 software.

#### Immunofluorescent staining of cells

Inducible nitric oxide synthase (iNOS, M1 marker) and arginase-1 (Arg-1, M2 marker) were used as markers to evaluate the polarization state of macrophages cultured on various specimens. After being fixed by 4% paraformaldehyde (PFA) for 20 min, permeabilized in 0.3% Triton-X for 10 min and blocked with 10% bovine serum albumin (BSA) solution for 2 h, the cells were treated with iNOS (Abcam, USA) and Arg-1 (Abcam, USA) primary antibody following the manufacturer’s instructions. Thereafter, the as-treated cells were incubated with Alexa Fluor 594 and 488 conjugated secondary antibody (Abcam, USA) for iNOS and Arg-1, respectively. Subsequently, the specimens were stained by 4′-6-diamidino-2-phenylindole (DAPI; Sigma, USA) for cell nuclei before the observation using a confocal laser microsope (Nikon, Japan).

#### Flow cytometry

To measure M1 and M2 phenotypes of macrophages, the flow cytometry was employed to determine the expression of CD86 (M1 marker) and CD206 (M2 marker), respectively. RAW264.7 cells were seeded on samples with a density of 60,000 cells per well in 24-well culture plates, and 5 well were collected as a sample. After being cultured for 1 and 5 days, the cells were digested and rinsed with PBS. The cells were incubated with FITC conjugated anti-mouse CD86 antibody (eBioscience, USA) diluted with PBS at a ratio of 1:50, and FITC conjugated anti-mouse CD206 antibody (eBioscience, USA) diluted with PBS at a ratio of 1:400 at 4 °C for 30 min in the dark. The RAW264.7 cells seeded on culture plates were used as a negative control. After being rinsed thrice with PBS, the labeled cells were determined by a flow cytometer (Beckman, USA).

#### Real time-quantitative polymerase chain reaction (RT-qRCR) and enzyme-linked immunosorbent assay (ELISA)

The gene expressions and cytokine productions of macrophages grown on various specimens were measured by RT-qPCR and ELISA, respectively. At day1 and day 5, the cells were lysed with Buffer MZ (Tiangen, USA) and the supernatants were collected and then filtered through 220 nm filter membranes. The schematic showing the measured method of ELISA and RT-PCR was showed in Fig. S1. For RT-qPCR assay, total RNA in the cell lysate was extracted using miRcute miRNA isolation kit (Tiangen, USA) and the complementary DNA was transcribed using FastQuant RT Kit (Tiangen, USA) accroding to the manufacturer’s instructions. The sequences of primers (Sangon Biotech, China) employed in this work were listed in Table S1. SYBR Green detection reagent (Bio-Rad, USA) was employed to amplify and detect cDNA targets on a real-time PCR detection system (CFX96, Bio-rad, USA). The mean cycle threshold (Ct) value was normalized to house-keeping gene GAPDH using the ΔΔCt method. For the ELISA test, the contents of tumor necrosis factor-α (TNF-α), interleukin-10 (IL-10), growth factors (BMP-2 and BMP-6) in the collected supernatants were measured by ELISA kits (Cusabio Biotech, China) accroding to the manufacturer’s instructions.

### Macrophage response to environmental pH and exogenous Mg^2+^

To study the modulatory role of environmental pH or exogenous Mg^2+^ on the macrophage response, the pH value and Mg^2+^ concentration of DMEM complete culture medium (pH at ~ 7.4 and Mg^2+^ concentration at ~ 19.2 μg/mL) was adjust to ~ 8.0 and 40 μg/mL through the addition of NaOH (1 mol/L) and MgCl_2_ (1 mol/L) solution, respectively. RAW 264.7 cells with a density of 60,000 cells per well were seeded in a 24-well plate. After being cultureding for 24 h, the culture medium was replaced by pH- or Mg^2+^-adjusted DMEM. After another 24 h, the viability of RAW 264.7 cells was measured by cell counting kit-8 (CCK-8) (Dojindo, Kumamoto, Japan) assay. The morphology and gene expressions of macrophages were evaluated as described in section 2.4.

### In vitro osteogenesis

#### Evaluation of macrophage-mediated osteogenesis

Approximately 99% of mouse genes have a homologue in the human genome [33]. Numerous research works have demonstrated the feasibility of mouse−human indirect co-culture systems [28, 34, 35]. Hence, the RAW 264.7 and SaOS-2 cells are employed to explore the osteogenic potential of the inflammatory micro-environment. SaOS-2 cells with a density of 30,000 cells per well were seeded in a 24-well plate and cultured with various CM. The proliferation of SaOS-2 cells was measured using the CCK-8 test. The maturation osteoblasts in response to various kinds of CM were characterized by alkaline phosphatase (ALP) activity on day 4, collagen (COL) production on day 10 and in vitro mineralization on day 14 as they indicate the early, middle and late stage of osteoblastic differentiation, respectively [36].

To evlatue the maturation of SaOS-2 cells at the early stage, the ALP activity was evaluated. The BCIP/NBT ALP color development kit was used to stain the cells for qualitative evaluation of ALP activity. For quantitative assessment of ALP activity, the cells were lysed with RIPA lysis buffer (Beyotime, China) at 4 °C for 15 min. The lysed supernatants were analyzed using ALP testing kits (Jiancheng, China) following the manufacturer’s instructions. The relative ALP activity was normalized to the protein content detected by BCA assay kits (Beyotime, China).

For COL production, after fixation with 4% PFA, the cells were stained with sirius red (Solarbio, USA) in darkness for 12 h and subsequently observed using an optical microscope (OM). Thereafter, the dyes were dissolved in a mixed solution composed of 50% NaOH (0.2 M in concentration) and 50% methanol for the measurements of the optical density (OD) values using a multimode reader at 520 nm.

For in vitro mineralization, the cells were fixed, stained with alizarin red (Solarbio, USA) and photographed under an OM. The dyes were dissolved in 10% cetypyridinum chloride. A multimode reader was employed to measure the OD values at 570 nm.

#### Effects of material extract medium (EM) on osteogenesis

Each specimen was immersed in 1 mL DMEM complete medium and incubated in incubator. After 24 h, the supernatants were collected and mixed with McCoy’s complete medium at a ratio of 1 to 6. Thereafter, SaOS-2 cells with a density of 30,000 cells per well were seeded in a 24-well plate and cultured with various EM. At pre-defined time points, the proliferation and differentiation of SaOS-2 cells in response to various CM were evaluated as described in section 2.6.1.

### Animals and surgery

The in vivo experiments were approved by the Ethics Committee of the Second Xiangya Hospital in Central South University. In this work, twelve male Sprague-Dawley rats (2-month-old, 250 ± 20 g) were used. The surgical procedure was carried out as previously reported [32]. Briefly, the surgical sites of anesthetized rats were shaved and sterilized. Thereafter, the defects were created in longitudinal axis of the femurs using an electric drill with a drill diamater of 2 mm. Subsequently, the sterilized implant was inserted into each pro-dirlled hole and the wound was sutured in layers. The surgery procedures were shown in Fig. S2. On day 7 or 28, the rats were sacrificed to get their bony tissues.

### Histological staining

The bony tissues were suscessively fixed in 4% PFA, decalcified by 10% ethylene diamine tetraacetic acid and embedded in polymethylmethacrylate. A microtome (Leica RM 2245, Germany) was employed to cut the specimens into slices with a thickness of 4 μm. Subsequently, the slices were stained with hematoxylin/eosin (H&E) and subsequently photogrphped using an OM.

For immunohistochemical staining, the slices were dewaxed, hydrated and treated with 3% H_2_O_2_ for 25 min. After being blocked for 30 min in 3% BSA, the slices were incubated with rabbit-anti-rat primary antibodies for CD11c (1:400), CCL24 (1:400) and BMP-2 (1:200) (Servicebio, China) overnight at 4 °C, respectively. Thereafter, each specimen was treated with goat-anti-rabbit HRP-conjugated secondary antibody for 50 min at room temperature. After that, the slices were stained with diaminobenzidine staining kit (Servicebio, China) and counterstained with hematoxylin. Finally, the images were obtained using an OM. The positive rate could be characterized by the areal density which represents the value of integral optical density (IOD)/ measured tissue area [37]. In this study, the quantitative results of immunohistochemistry for CD11c, CCL24 and BMP-2 were determined based on IOD/Area using Image-Pro Plus software.

### Statistical analysis

Each experiment was repeated four times in this work. The results were expressed as the mean value ± standard deviation (SD). The data analysis was performed following Student’s t-test. The level of significant difference among different groups was defined and noted as * *p* < 0.05 and ** *p* < 0.01.

## Results

### Microstructure and surface analysis

**Fig. 1:**
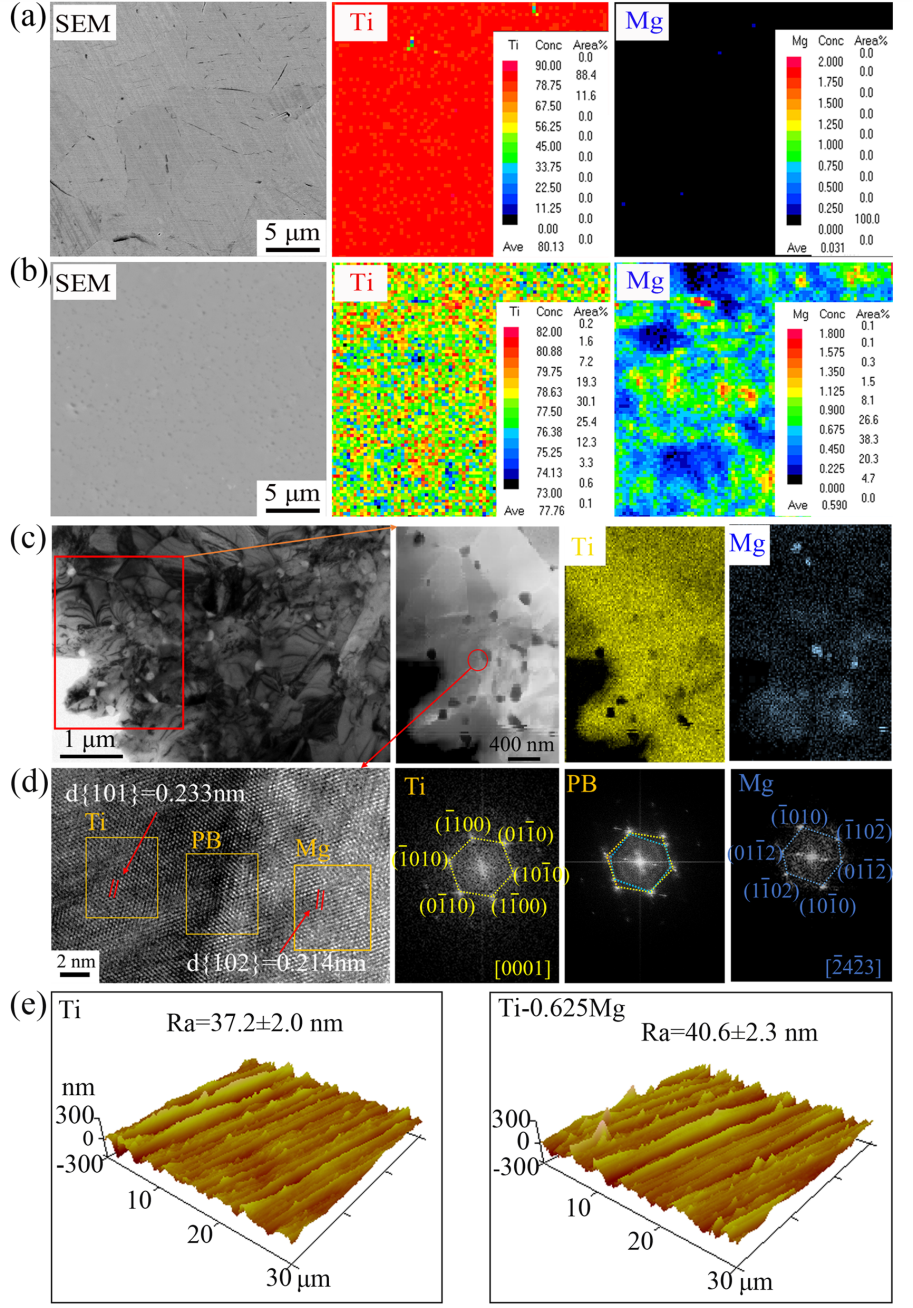
EPMA mapping analysis of **a** Ti and **b** Ti-0.625 Mg alloy; **c** The microstructure of the Ti-0.625 Mg alloy characterized by TEM and EDX elemental mapping; **d** high resolution (HR)-TEM image showing the phase boundary (PB) between Ti and Mg and corresponding selected area electron diffraction (SAED) patterns; **e** the surface roughness of Ti and Ti-0.625 Mg evaluated by AFM

As shown in Fig. 1a and b, Mg is distributed almost uniformly in the Ti-0.625 Mg alloy, while Mg was not detected in CP-Ti. The microstructure of the Ti-0.625 Mg alloy was characterized in-depth by TEM. As shown in Fig. 1c, sub-micron sized Mg particles with a mean diameter of 260.8 ± 27.2 nm were found to be homogeneously distributed at the boundaries of Ti grains. The average inter-particle spacing was measured to be around 462.7 nm. The result of EDX elemental mapping showed the presence of Mg-enriched particles, of which the locations were well overlapped with those of the Mg phase shown in TEM image. Several pores were also noticed in the Ti matrix due to Mg dissolution during the TEM specimen preparation process. Besides those bright Mg-enriched particles, Mg signals were detected throughout the Ti matrix. This indicated that partial Mg still existed as a solid solute the in Ti matrix. It is well known that both pure Ti and Mg have hexagonal crystal structure at room temperature [38]. In this work, two sets of SAED patterns were found to correspond to Ti and Mg, respectively. Their orientation relationships can be expressed as α-Ti (− 1100)//Mg (− 1010), α-Ti (1-100)//Mg (10 -10). The theoretical interplanar spacing of d{101}α-Ti and d{102}Mg are known to be 0.224 nm (PDF#44-1294) and 0.190 nm (PDF#35-0821), respectively. According to the SEAD patterns, the lattice parameter of d{101}α-Ti or d{102}Mg from SEAD patterns was found to be 0.233 nm or 0.214 nm. Using SAED images and XRD techniques, Magdalena et al. [39] demonstrated that the lattice parameter increase of the SnO2/Sb phase was related to the geometrical distortion of the lattice due to the replacement of Sn by Sb. These results indicated the mutual dissolution between Mg and Ti. To reduce the effect of surface roughness, the surface of both CP-Ti and the Ti-0.625 Mg alloy was ground to 1500 mesh. As shown in Fig. 1e, the surface roughness values of Ti and Ti-0.625 Mg after polishing were measured to be 37.2 ± 2.0 and 40.6 ± 2.3 nm, respectively.

### Evaluation of Mg degradation in the Ti-0.625 Mg alloy

As listed in Table 1, the pH value and Mg^2+^ concentration in Ti group were relatively stable on various days as Ti is known to be non-degradable. The results revealed the original levels of the alkalinity and Mg^2+^ concentration of DMEM. In the Ti-0.625 Mg group, the pH value and Mg^2+^ concentration declined continuously from day 1 to 5. This suggests that the degradation rate of Mg in the Ti-0.625 Mg alloy gradually reduced after immersion for 5 days.

**Table 1:** The changes of pH values and Mg^2+^ concentrations in DMEM after various specimens were immersed for 1, 3 and 5 days

Group	Ti (Mean ± SD)		Ti-0.625 Mg (Mean ± SD)	
	pH value	Mg^2+^ concentration (mg/L)	pH value	Mg^2+^ concentration (mg/L)
Day 1	7.43 ± 0.06	19.23 ± 0.24	8.03 ± 0.11	39.70 ± 0.41
Day 3	7.45 ± 0.08	19.35 ± 0.23	7.79 ± 0.08*	30.12 ± 0.35**
Day 5	7.45 ± 0.05	19.30 ± 0.26	7.67 ± 0.07*	26.96 ± 0.57**

*p < 0.05 and **p < 0.01 compared to Day 1

### Macrophage response to the Ti-0.625 Mg alloy

#### Cell proliferation and morphology

**Fig. 2:**
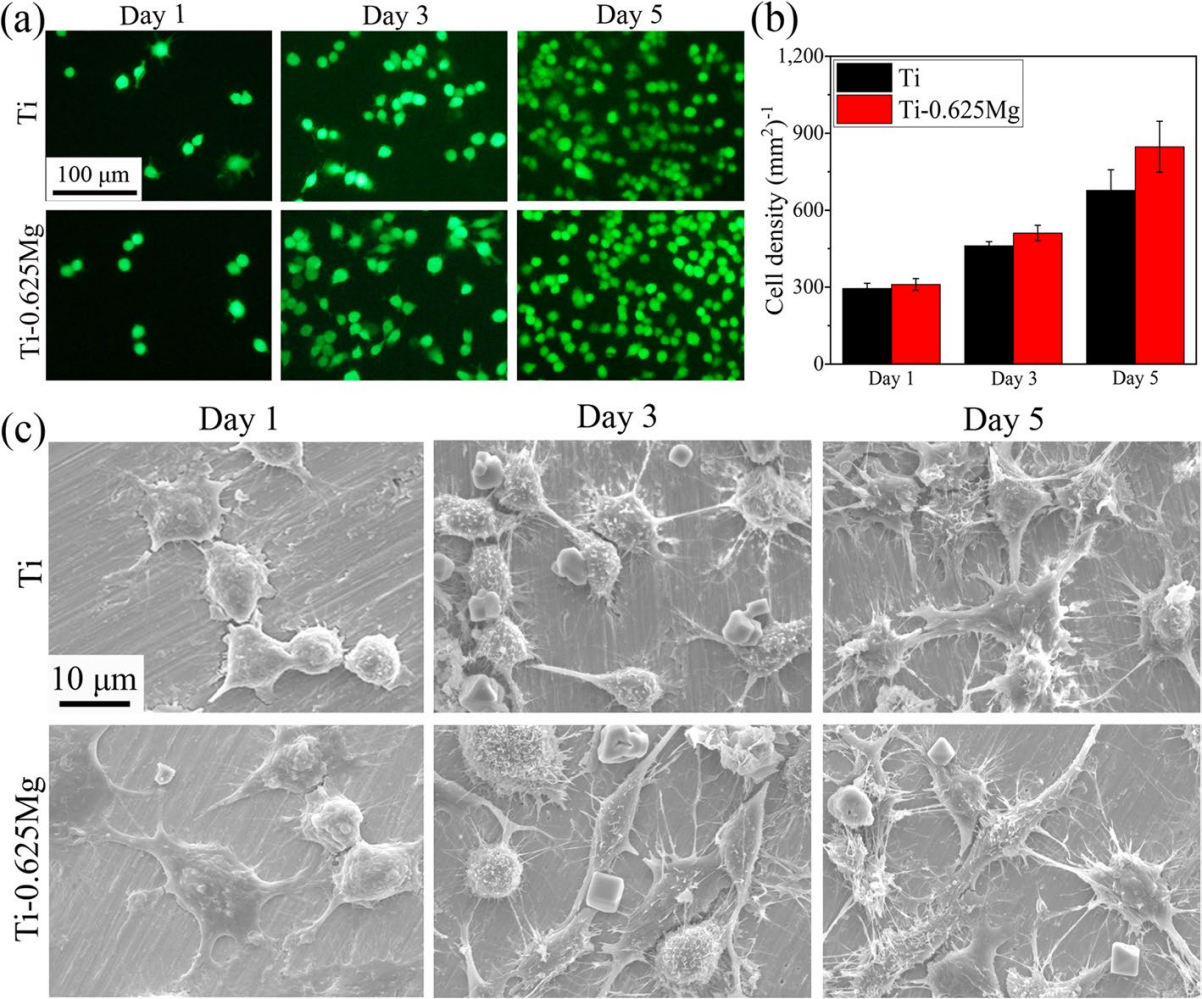
**a** The presence of live macrophages on Ti or Ti-0.625 Mg surfaces evaluated by calcein-AM staining and **b** quantitative results of cell numbers as well as **c** cell morphology revealed by SEM on day 1, 3 and 5. *p < 0.05 and **p < 0.01 compared to the Ti

The proliferation of macrophages grown on the Ti and Ti-0.625 Mg surfaces was assessed using calcein-AM staining for live cells and quantified by Image-Pro Plus 6.0 software. As shown in Fig. 2a and b, both the Ti and Ti-0.625 Mg surfaces supported the growth and proliferation of macrophages. Moreover, the proliferation rates of macrophages were comparable in Ti and Ti-0.625 Mg groups. The morphology of macrophages in response to Ti or Ti-0.625 Mg surfaces for various durations was shown in Fig. 2c. On day 1, the extension of pseudopodia was observed for macrophages grown on both Ti and Ti-0.625 Mg surface. The cells exhibited a more flattened morphology on the Ti-0.625 Mg surface than on the control surface. On day 3, the formation of filopodia was found to be more obvious on the Ti-0.625 Mg surface. Moreover, the cells grown on the Ti surface exhibited a stellate morphology, while those in the Ti-0.625 Mg group were either spherical or fusiform. Till day 5, the cells were more extended and stellate in shape on the Ti surface than those cultured for 3 days. On the Ti-0.625 Mg surface, the phenomenon of cell elongation was also more significant than that on day 3.

#### Macrophage polarization

**Fig. 3:**
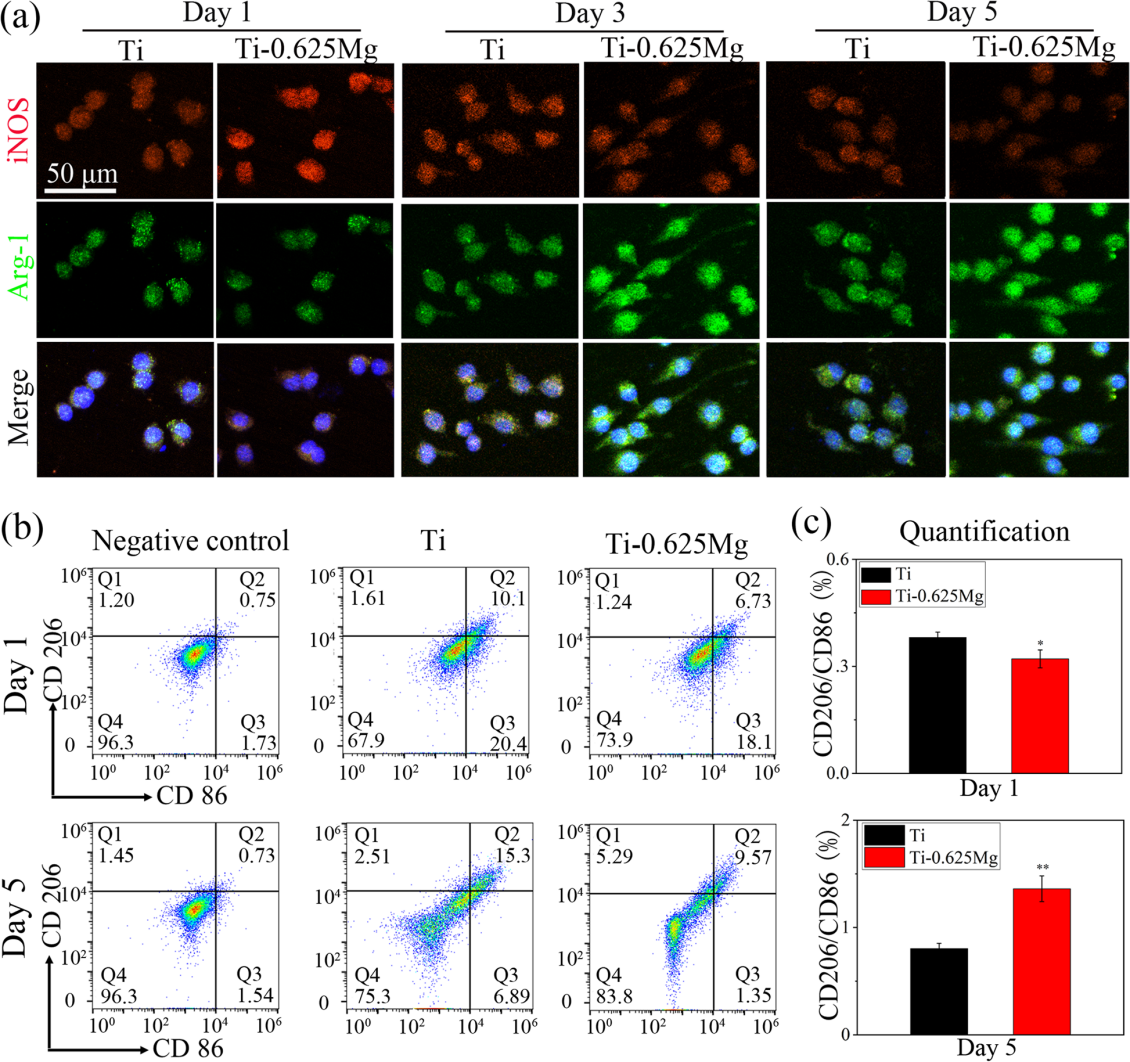
**a** Immunofluorescent staining images showing the expression levels of iNOS (M1 marker, in red) and Arg-1 (M2 markers, in green) in macrophages grown on specimen surfaces for day 1, 3 and 5, and the nuclei of cells were stained with DAPI (in blue), **b** Flow cytometry analysis of macrophage marker expression for day 1 and 5, **c** The ratio of M2 (CD206) to M1 (CD86) macrophages on Ti and Ti-0.625 Mg surfaces. *p < 0.05 and **p < 0.01 compared to the Ti

The activation process of macrophages cultured on the Ti-0.625 Mg surface was evaluated at both gene- and protein-level by immunofluorescent staining, flow cytometry, RT-PCR and ELISA tests. As shown in Fig. 3a, the expressions levels of iNOS and Arg-1 in macrophages grown on the Ti surface did not change significantly from day 1 to 5, even though slightly down-regulated iNOS expression was noticed on day 5 compared to that on day 1 and 3. However, the expression levels of iNOS and Arg-1 gradually decreased and increased in the Ti-0.625 Mg group from day 1 to 5, respectively. Compared to those in the Ti group, macrophages grown on the Ti-0.625 Mg surface expressed higher iNOS level on day 1 and lower iNOS level on day 5. The expression level of Arg-1 was higher in the Ti-0.625 Mg group than that in the Ti group from day 3 to 5.

As shown in Fig. 3b, the proportion of the M1 phenotype and M2 phenotype of macrophages was measured by flow cytometry. The ratio of the fluorescence intensity of CD206 (M2 marker) to that of CD86 (M1marker) was showed in Fig. 3c. For 1 day, the ratio of CD206/CD86 of Ti and Ti-0.625 Mg group is less than 1, and the value of the Ti-0.625 Mg group is significantly lower than that of the Ti group. These results indicated that Ti and Ti-0.625 Mg induced more macrophages to the M1 phenotype than to the M2 phenotype, and Ti-0625 Mg induced much more M1 macrophages and much fewer M2 macrophages than Ti. For 5 days, the ratio of CD206/CD86 of Ti group is less than 1, and CD206/CD86 of Ti-0.625 Mg group is more than 1. These results indicated that Ti-0625 Mg induced much fewer M1 macrophages and much more M2 macrophages than Ti.

**Fig. 4:**
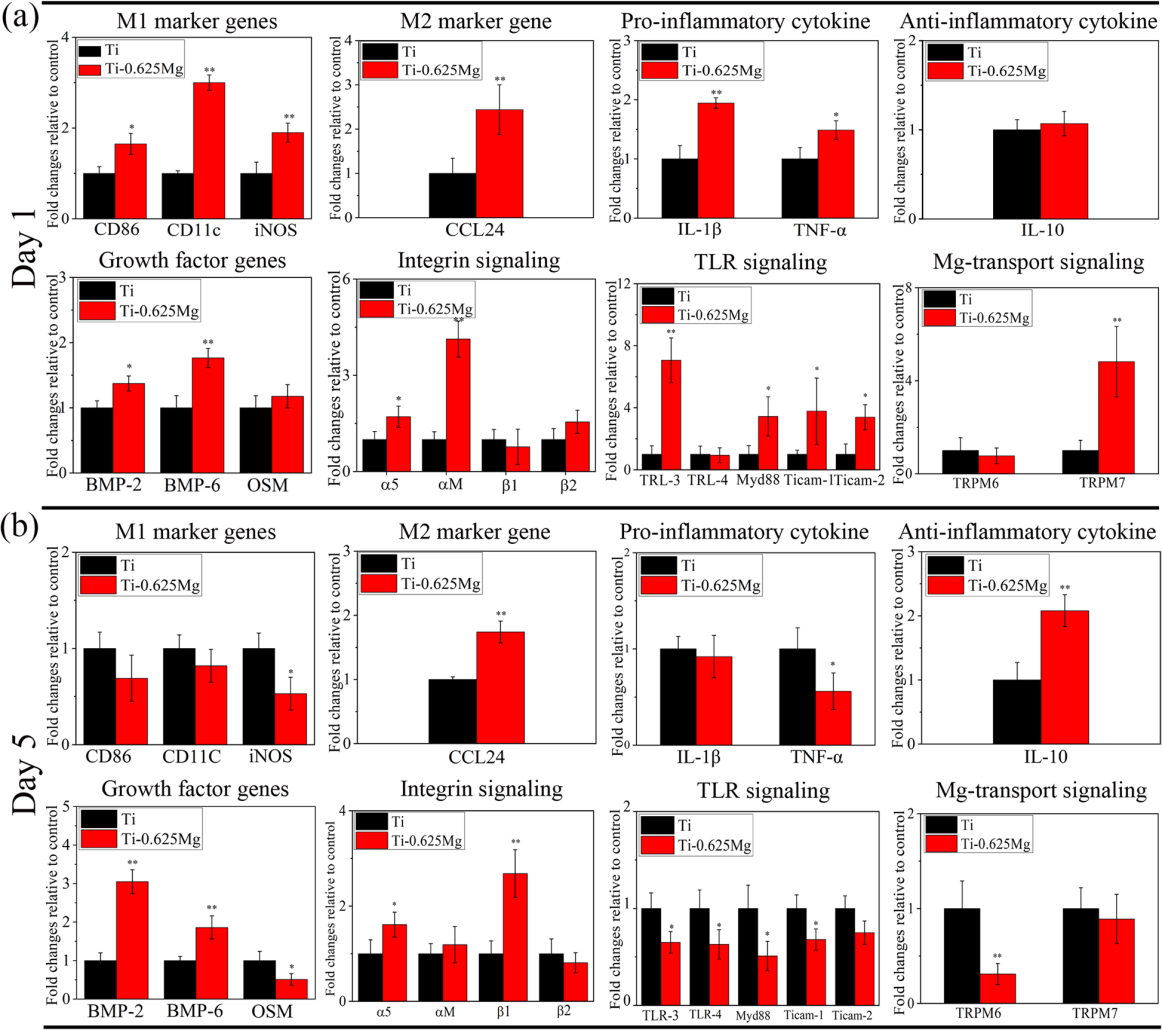
The expressions of inflammation-related genes in macrophages grown on specimen surfaces for **a** 1 and **b** 5 days. * *p* < 0.05 and ** *p* < 0.01 compared to the Ti

As shown in Fig. 4a, the macrophages cultured on the Ti-0.625 Mg surface expressed higher levels of M1 (CD86, CD11c and iNOS) and M2 (CCL24) marker gene, pro-inflammatory cytokine (IL-1β and TNF-α) and growth factor (BMP-2 and BMP-6) genes than those grown on the Ti surface on day 1. Furthermore, the gene expressions related to integrin (α5 and αM), toll-like receptor (TLR; TLR-3, Myd88, Ticam-1 and Ticam-2) and Mg-transport (TRPM7) signaling were also up-regulated in the Ti-0.625 Mg group on day 1. On day 5, the gene expressions of M2 marker gene (CCL-24), anti-inflammatory cytokine (IL-10), growth factors (BMP-2 and BMP-6) were up-regulated in macrophages grown on the Ti-0.625 Mg surface, while those of iNOS, TNF-α and OSM (growth factor) were down-regulated. Compared with those in the Ti group on day 5, the gene expressions related to integrin (α5 and β1) signaling in the Ti-0.625 Mg group were up-regulated, while those related to TLR (TLR-3, TLR-4, Myd88 and Ticam-1) and Mg-transport (TRPM6) signaling were down-regulated.

**Fig. 5:**
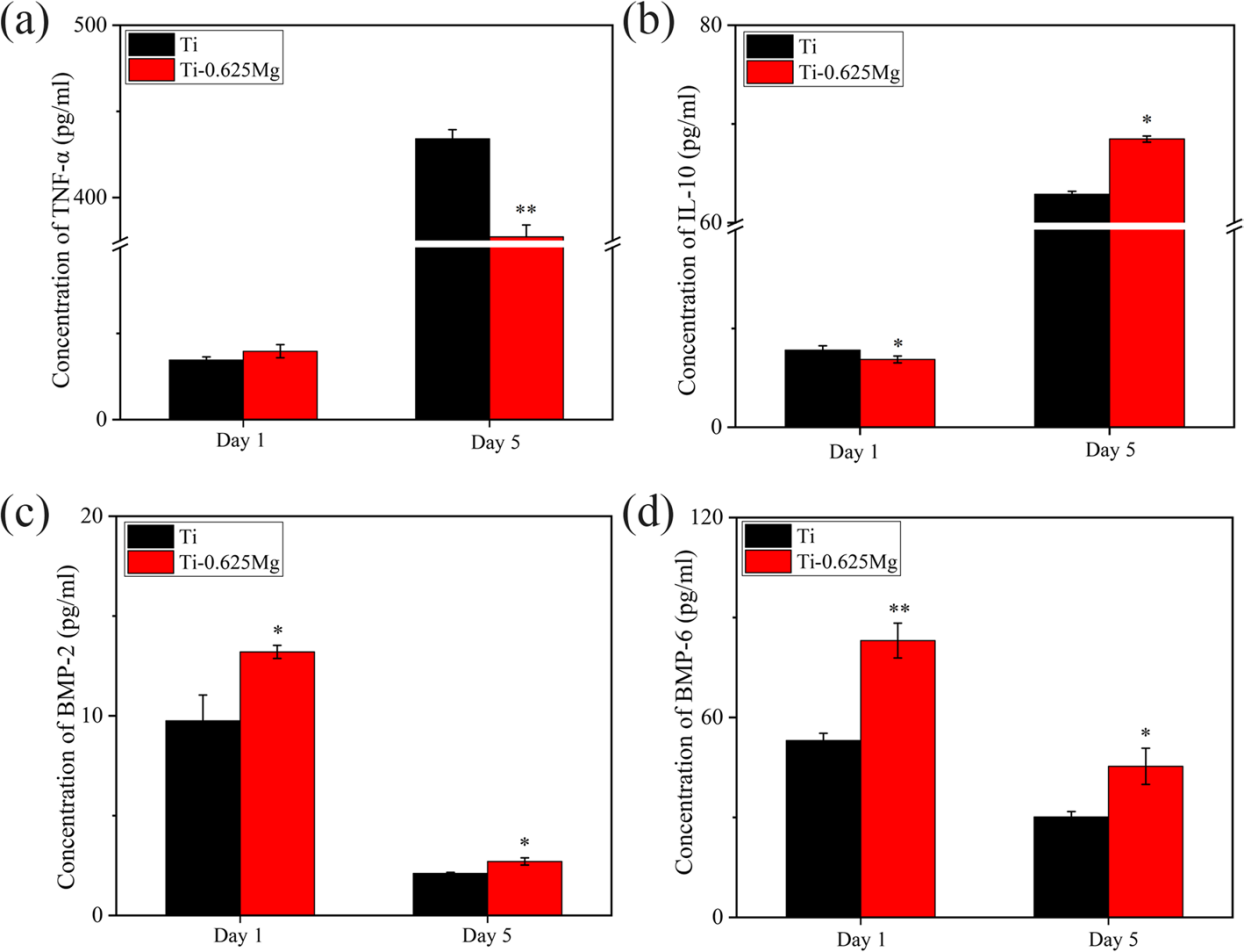
The production of **a** TNF-α, **b** IL-10, **c** BMP-2 and **d** BMP-6 by macrophages grown on various specimen surfaces for 1 and 5 days. * *p* < 0.05 and ** *p* < 0.01 compared to the Ti

The expressions of cytokines (TNF-α and IL-10) and growth factors (BMP-2 and BMP-6) were also evaluated by ELISA. As shown in Fig. 5, the macrophages in both groups secreted higher levels of TNF-α and IL-10 on day 5 and higher levels of BMP-2 and BMP-6 on day 1. The production of TNF-α in the Ti-0.625 Mg group was higher than that in the Ti group on day 1, but lower on day 5. On the contrary, the secretion of IL-10 in the Ti-0.625 Mg group was reduced on day 1 and enhanced on day 5 compared to that in the Ti group. The productions of BMP-2 and BMP-6 were enhanced in the Ti-0.625 Mg group on both day 1 and 5.

### Macrophage response to environmental pH and exogenous Mg^2+^

**Fig. 6:**
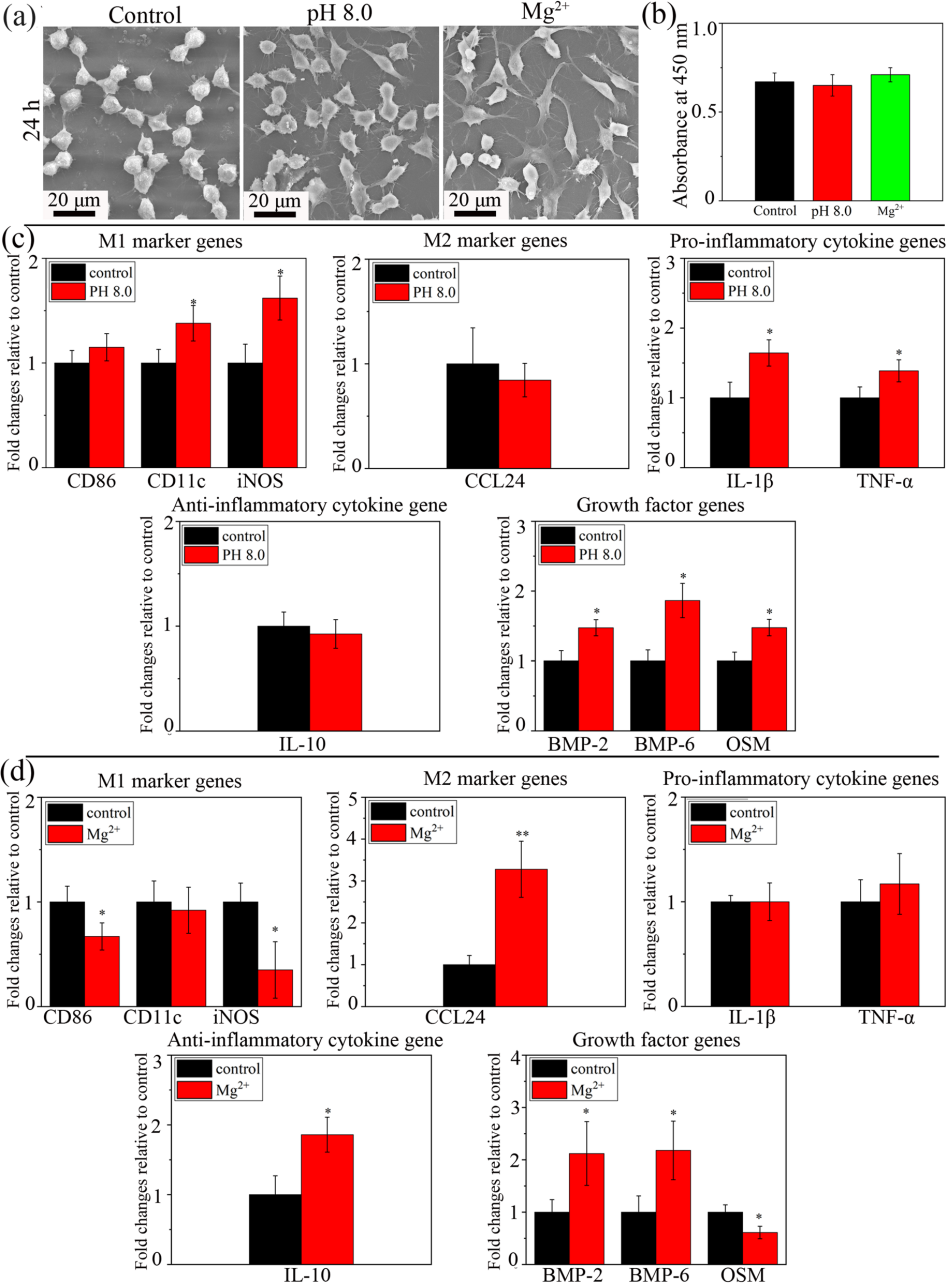
Effects of environmental pH (~ 8.0) and exogenous Mg^2+^ (~ 40 μg/ml) on macrophage behavior compared to those under normal culture medium condition (pH at ~ 7.4 and Mg^2+^ concentration at ~ 19.2 μg/mL): cell **a** morphology and **b** viability; inflammation-related gene expressions in macrophages in response to **c** environmental pH and **d** exogenous Mg^2+^. * *p* < 0.05 and ** *p* < 0.01 compared to the control

In order to clarify the individual role played by the environmental pH (~ 8.0) or Mg^2+^ (~ 40 μg/ml) in modulating macrophage response, the pH and Mg^2+^ concentration of DMEM complete culture medium (pH at ~ 7.4 and the Mg^2+^ concentration at ~ 19.2 μg/mL) employed for macrophage cultivation were adjusted through the additional supplement of NaOH and MgCl_2_ solutions, respectively. As shown in Fig. 6a, the macrophages were mainly roundish in the control group, polygonal in the pH 8.0 group and elongated in the Mg^2+^ group. The levels of macrophage viability were comparable in these three groups (Fig. 6b). Compared with those in the control group (pH at ~ 7.4), the gene expressions of M1 marker genes (CD11c and iNOS), pro-inflammatory cytokines (IL-1β and TNF-α) and growth factors (BMP-2, BMP-6 and OSM) were up-regulated in the pH 8.0 group. Compared to with those in the control group (Mg^2+^ concentration at ~ 19.2 μg/mL), the expressions of M2 marker gene (CCL24), anti-inflammatory cytokine (IL-10) and growth factor (BMP-2 and BMP-6) genes were up-regulated in the Mg^2+^ group (Mg^2+^ concentration at ~ 40 μg/ml), while the gene expressions of M1 marker gene (CD86 and iNOS) and growth factor OSM were down-regulated.

### In vitro osteogenesis

#### Macrophage-mediated osteogenesis

**Fig. 7:**
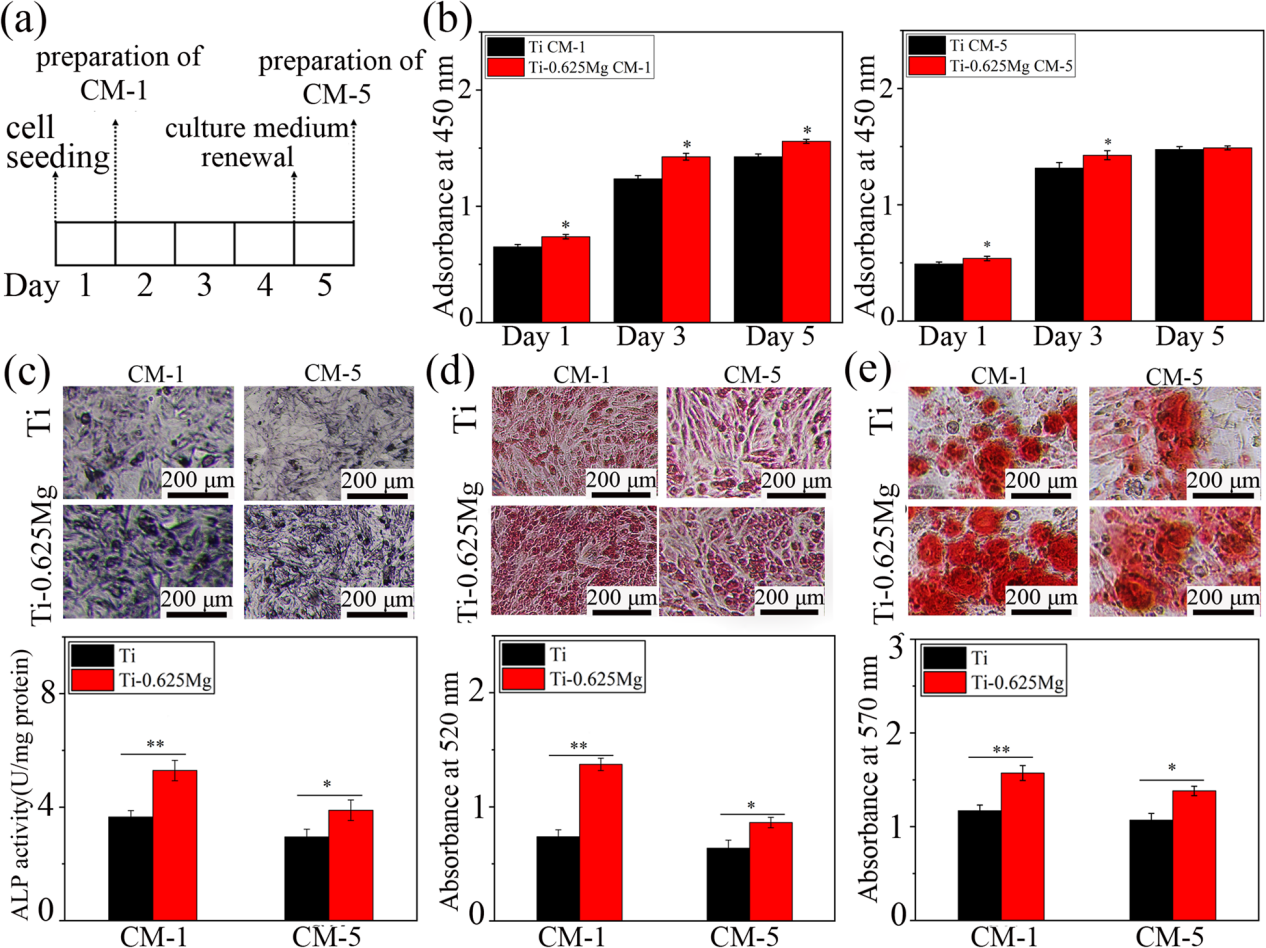
**a** The schematic showing the preparation method of CM-1 and CM-5; **b** the proliferation on day 1, 3 and 5, **c** ALP activity on day 4, **d** COL production on day 10 and **e** in vitro mineralization on day 14 of SaOS-2 cells in response to various kinds of CM. * *p* < 0.05 and ** *p* < 0.01 compared to the Ti

**Fig. 8:**
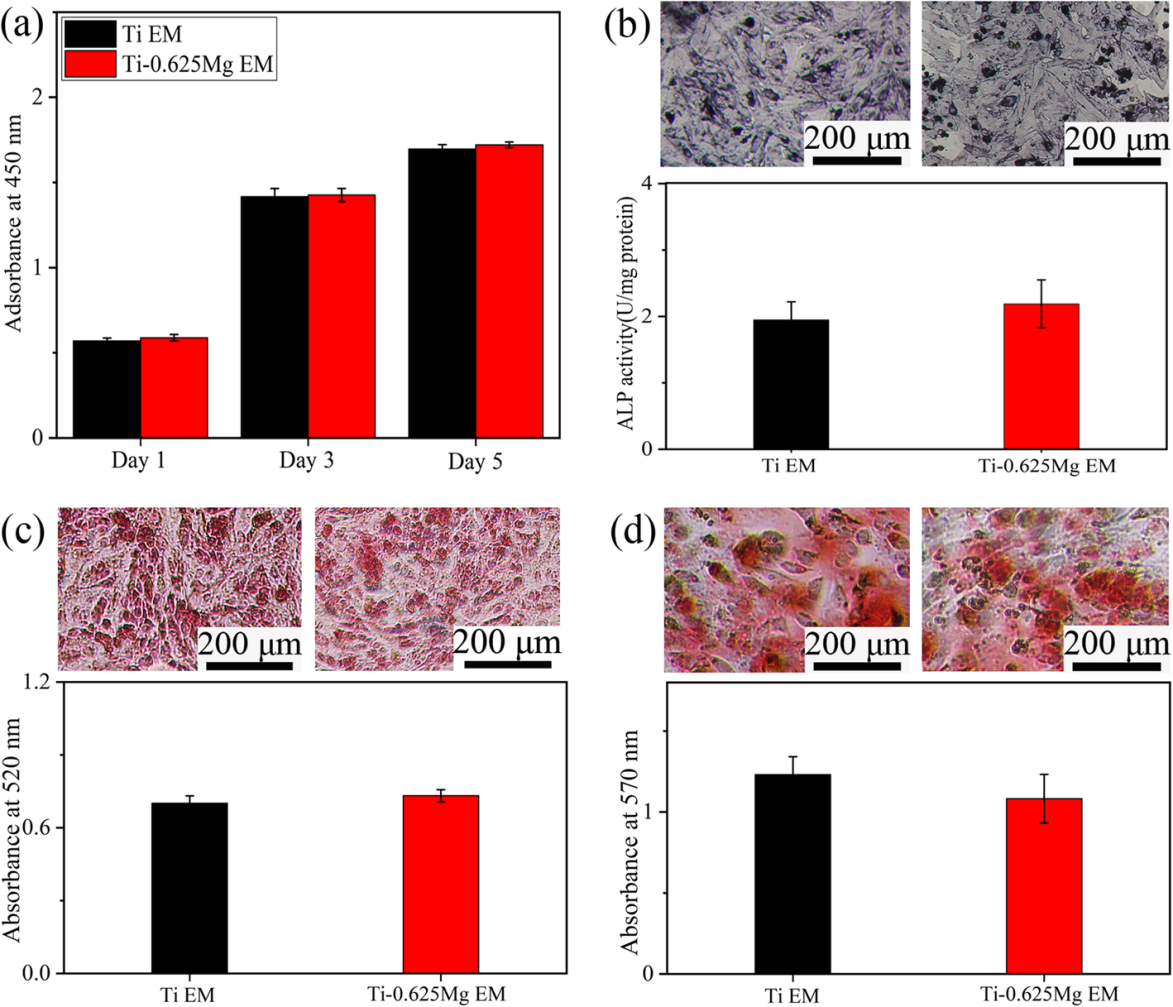
**a** The proliferation on day 1, 3 and 5, **b** ALP activity on day 4, **c** COL production on day 10 and **d** in vitro mineralization on day 14 of SaOS-2 cells in response to Ti and Ti-0.625 Mg extract medium (EM). *p < 0.05 and **p < 0.01 compared to the Ti EM

To investigate the relationship between macrophage phenotypes and their osteogenic potential, various kinds of CM were prepared according to the procedures presented in Fig. 7a. The proliferation and differentiation of SaOS-2 cells grown in various CM were studied. As shown in Fig. 7b, Ti-0.625 Mg CM-1 promoted SaOS-2 cell proliferation on day 1, 3 and 5 compared with Ti CM-1, while Ti-0.625 Mg CM-5 enhanced the proliferation of SaOS-2 cells on day 1 and 3 compared with Ti CM-5. For cell differentiation, CM-1 and CM-5 in the Ti-0.625 Mg group enhanced the ALP activity, COL production and in vitro mineralization of SaOS-2 cells compared with CM-1 and CM-5 in the Ti group, respectively (Fig. 7c–e). As shown in Fig. 8, the proliferation, ALP activity, COL production and in vitro mineralization of SaOS-2 cells were comparable in both Ti and Ti-0.625 Mg EM groups. It indicated that the extract medium from the Ti-0.625 Mg alloy had no effect on the proliferation and differentiation of SaOS-2 cells due to the lower Mg^2+^ concentration.

### Histological evaluation of inflammation and osteogenesis in vivo

**Fig. 9:**
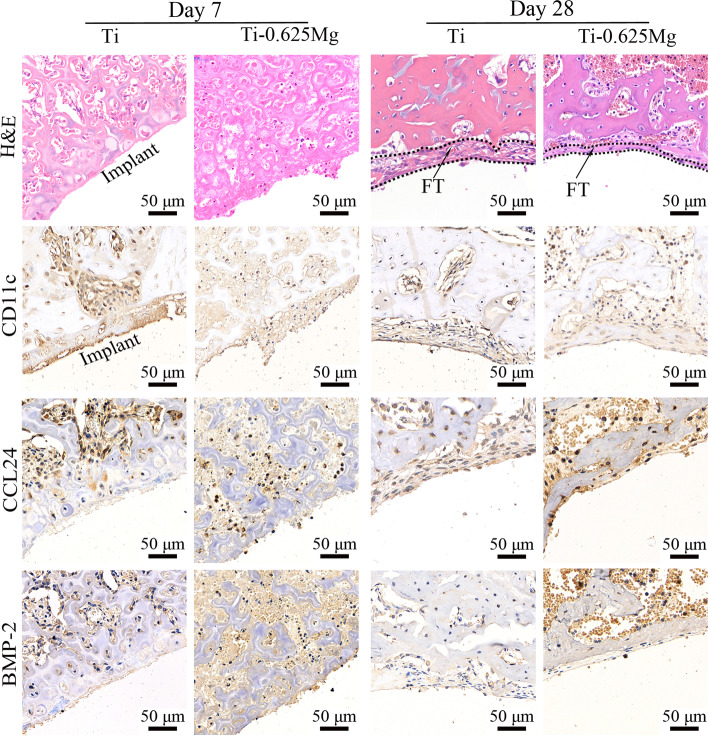
The histological images of H&E and immunohistochemical staining of CD11c, CCL24 and BMP-2 expressions at bone-material interfaces after implantation for 7 and 28 days (FT: fibrous tissue; for immunohistochemical staining, yellow indicates expression)

**Fig. 10:**
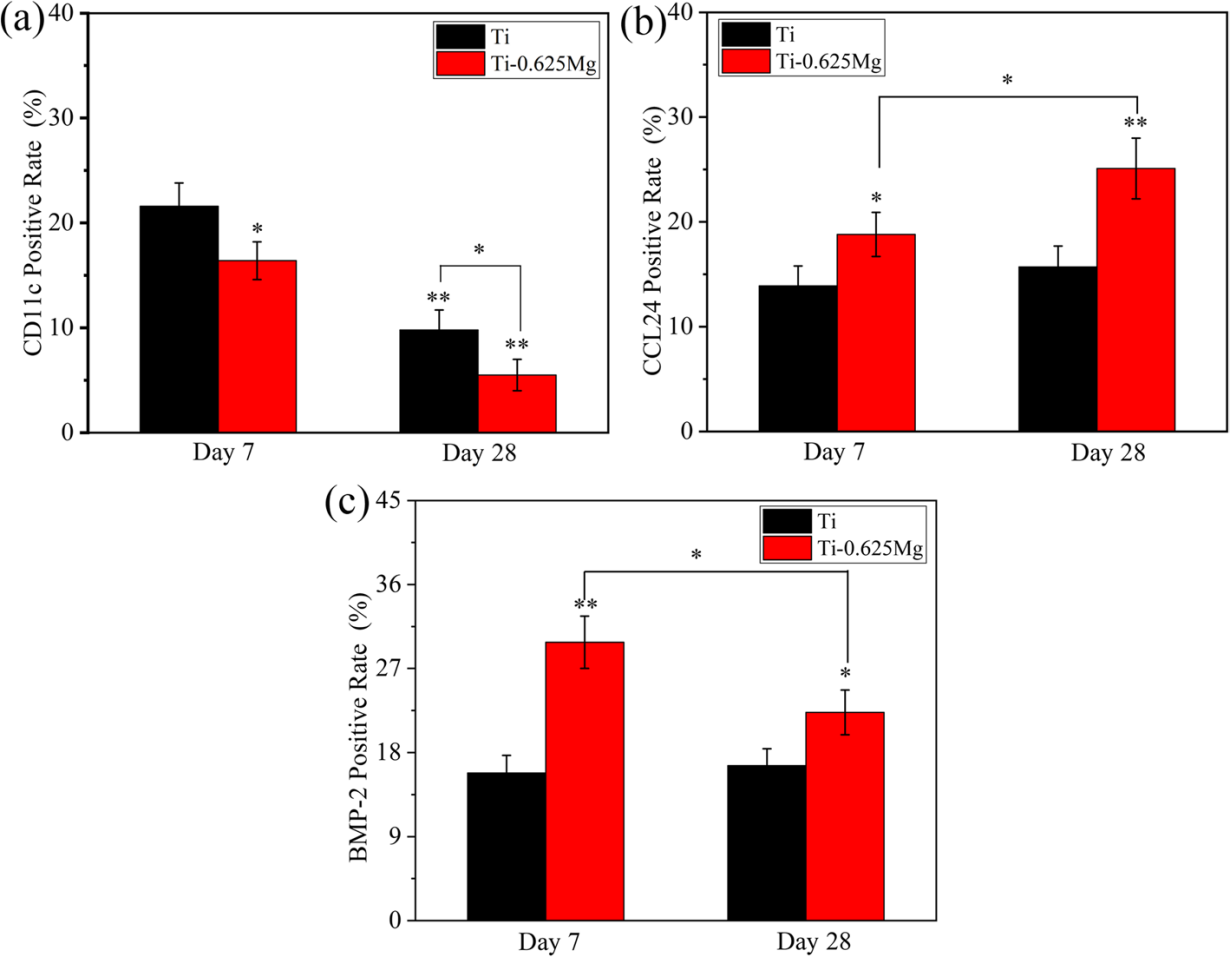
Quantitative analysis of positively stained areas using Image-Pro Plus software. *P < 0.05, **P < 0.01 versus the control group

Generally, fibrous cells have layered and shuttle shapes, whereas new bone exhibits holes (Haversian canal) and trabecular bone structures. In the previous work, we have demonstrated that the Ti-0.625 Mg alloy has better in vivo osteogenetic properties than pure Ti [32], but the relationship between inflammation and osteogenic properties was not discussed. As shown in Figs. 9 and 10, the fibrous tissue was found in the Ti and Ti-0.625 Mg groups according to the H&E staining images for day 28. The thickness of the fibrous tissue layer in the Ti group was significantly thicker than that in than Ti-0.625 Mg group. As a pro-inflammatory marker, the expression levels of CD11c were higher on day 7 in both the Ti and Ti-0.625 Mg groups than those on day 28. Moreover, the Ti-0.625 Mg group exhibited reduced CD11c expressions compared with the Ti group after implantation for 7 and 28 days. Compared with those in the Ti group, the expressions of CCL24 and BMP-2 were found to be enhanced in the Ti-0.625 Mg group on both day 7 and 28. In the Ti-0.625 Mg group, the expression level of CCL24 was found to be higher, while the expression of BMP-2 was lower on day 28 than those on day 7.

## Discussion

### Material microstructure and properties

The microstructure of the Ti-0.625 Mg alloy is directly dependent on the fabrication process. During MA, the high-energy milling process could drive Mg to dissolve into the Ti matrix [40]. It was confirmed in our previous work that the XRD patterns of Mg and Ti mixed powders after milling for 8 h exhibited no peak corresponding to Mg [32]. SPS is a fast sintering process in which powder is densified into bulk alloy by strong current under high pressure [41]. In the subsequent SPS process, partial Mg could be re-precipitated at the boundaries of Ti grains due to the fairly low solubility of Mg in Ti (~ 0.15 wt.%), while the rest of it still existed in the Ti matrix in the form of a solid solute [42]. This is evidenced by the EDX elemental mapping result of Mg in the Ti-0.625 Mg alloy presented in Fig. 1a.

The degradation rate of Mg in the Ti-0.625 Mg alloy gradually decreased due to Mg consumption, evidenced by the changes of environmental pH and Mg^2+^ concentrations listed in Table 1. The degradation of re-precipitated Mg particles at Ti grain boundaries is related to the initial significant increase in the environmental pH and Mg^2+^ concentration. However, the solid solute Mg in the Ti matrix could dissolve slowly and contribute to long-term release of Mg^2+^ and OH^−^. The degradation behavior of Mg in the Ti-0.625 Mg alloy was found to be dynamic. Thus, it could be employed as a material model to study the phased modulatory roles of Mg degradation on macrophage polarization. In addition, the polished Ti and Ti-0.625 Mg discs exhibited comparable surface roughness (Fig. 1c) and wettability (water contact angle at 55 ~ 57°) [32]. Therefore, the influence of differences in material surface roughness and wettability on the modulating macrophage response could be largely minimized.

### Macrophage activation in response to the Ti-0.625 Mg alloy

The activating process of macrophages grown on the Ti-0.625 Mg surface was found to be dynamic. The Ti-0.625 Mg alloy tended to polarize macrophages to their pro-inflammatory M1 phenotype on day 1. However, different from that of typically polarized M1 macrophages, the gene expression of CCL24 (M2 marker gene) was also up-regulated in the Ti-0.625 Mg group. Thus, the phenotype of macrophages activated by the Ti-0.625 Mg alloy on day 1 could be termed as M1 [16]. On day 5, the phenotype of macrophages grown on the Ti-0.625 Mg surface for 5 days could be termed as M2. This phenomenon was also confirmed at the protein level (Fig. 5). Consistently, the macrophages grown on the Ti-0.625 Mg surface secreted larger amount of TNF-α and lower amount of IL-10 on day 1 than those in the Ti group (Fig. 5a and b). However, opposite results were noticed on day 5. Even though the expression of IL-10 was not down-regulated at gene level in the Ti-0.625 Mg group on day 1, it was found to be down-regulated at the protein level. This is probably because that the RT-PCR and ELISA tests were conducted at the same time point and the change in gene expression is commonly earlier than that of protein secretion. Together, the results suggested that the macrophages underwent sequential activation of M1 and M2 phenotypes in response to Mg degradation in the Ti-0.625 Mg alloy. The sequential activation of phenotypes in macrophages by the Ti-0.625 Mg alloy may be attributed to the dynamic degradation behavior of Mg. It has been reported that the rapid corrosion of Mg-based materials could induce excessive inflammatory response, while various surface coatings designed to suppress Mg corrosion may attenuate proinflammatory gene expressions [43, 44].

The individual role played by environmental pH or Mg^2+^ in the activation of macrophage was investigated. The cytoskeletal morphology of macrophages is highly associated with their phenotypes [45]. Elongated cytoskeleton is commonly observed for M2 macrophages, while the roundish or polygonal shape is typical for M1 macrophages [28, 46]. The results of cell morphologies presented in Fig. 6a were consistent with previous findings. The morphological features of macrophages grown on the Ti-0.625 Mg surface underwent a changing process from a roundish or polygonal shape to a fusiform shape (Fig. 2c). The RT-PCR results showed that the gene expressions of macrophages cultured in pH-adjusted DMEM (pH at ~ 8.0) were more similar to those in the Ti-0.625 Mg group on day 1, while those of macrophages grown in Mg^2+^-adjusted DMEM (Mg^2+^ concentration at ~ 40 μg/mL) were close to those in the Ti-0.625 Mg group on day 5 (Fig. 6). These results indicated that the change in environmental pH played a more important role than Mg^2+^ for the Ti-0.625 Mg alloy in inducing the polarization of macrophages on day 1, while the released Mg^2+^ was more responsible for the macrophage activation on day 5. It is probably because that the culture medium renewal process could eliminate the increased environmental alkalinity, while Mg^2+^ continuously accumulated within macrophages. Moreover, it is noticed that the gene expression of CCL24 (M2 marker gene), which was not up-regulated in macrophages grown in pH-adjusted DMEM (Fig. 6c), increased in the Ti-0.625 Mg group on day 1 (Fig. 4a). This indicates that environmental pH and Mg^2+^ exhibited a coupling effect in inducing macrophage activation. Generally, it is considered that the sequential activation of macrophage phenotypes is attributed to the dynamic degradation behavior of Mg in the Ti-0.625 Mg alloy.

**Fig. 11:**
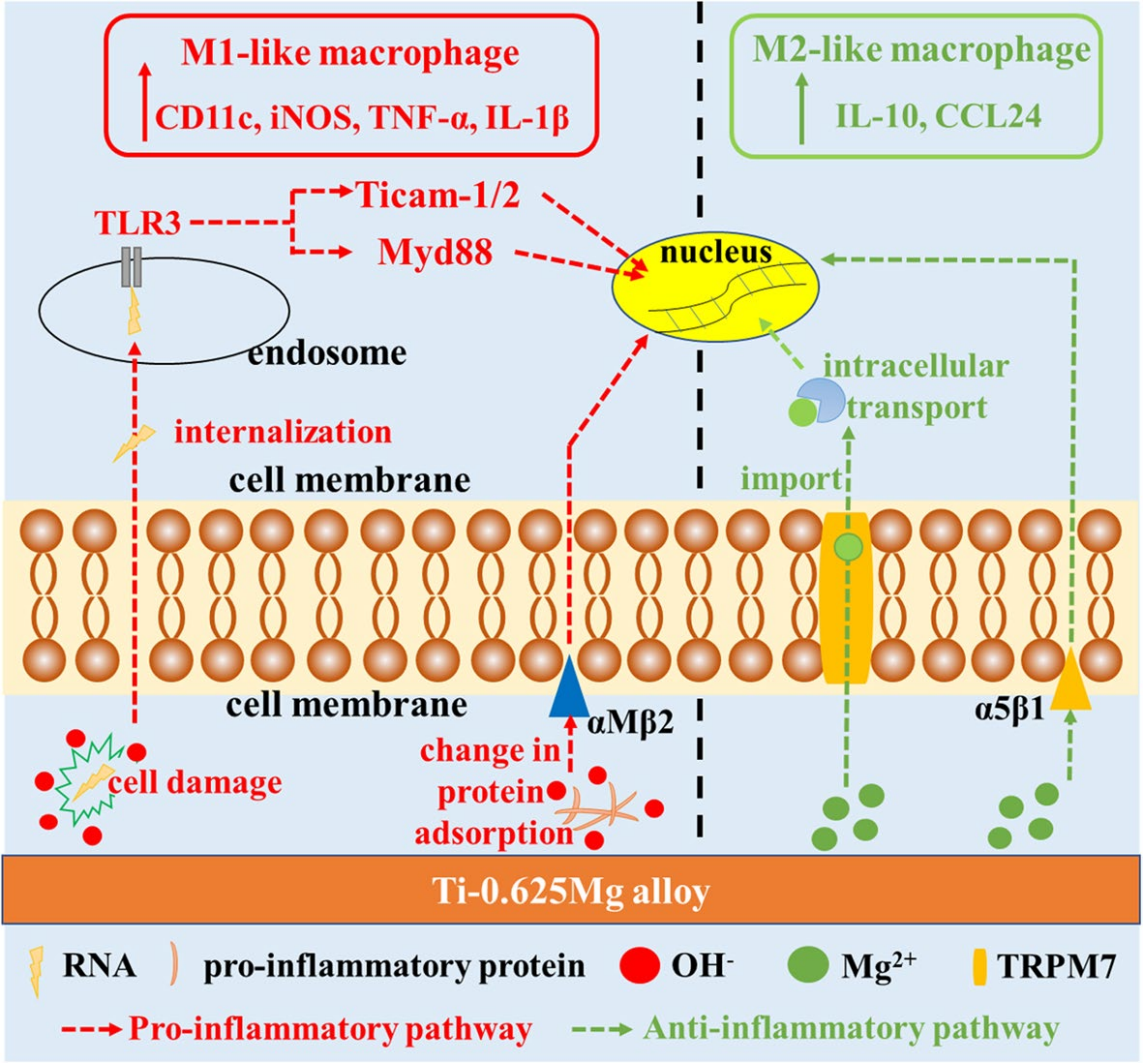
The schematic diagram showing the signaling pathways involved in the Ti-0.625 Mg alloy-mediated macrophage activation

To investigate the pathways involved in the Ti-0.625 Mg alloy-induced macrophage activation, the gene expressions associated with integrin, TLR and Mg-transport signaling in response to the Ti-0.625 Mg alloy were studied. As transmembrane receptors, integrins can recognize specific amino acid sequences in extracellular matrix (ECM) and guide cell-ECM interactions [47]. The previous work reported that αM could bind to fibrinogen which is a well-known pro-inflammatory protein to activate M1 phenotype in macrophages [47, 48]. Thus, it is speculated that the increase in environmental pH (~ 8.03) could modulate the types, amounts or conformations of proteins such as fibrinogen adsorbed on material surface and activate macrophages in a pro-inflammatory manner through the up-regulation of αM. TLRs are capable of recognizing invaders and damaged cell debris [49]. TLR-3 is known to be presenting in cell endosome and can recognize self-RNA originated from damaged cells [50]. Hence, it is inferred that the high alkalinity (pH at ~ 8.03) caused by Mg degradation in the Ti-Mg alloy on day 1 might lead to a certain degree of cell damage, which could subsequently be recognized by TLR-3 within macrophages. The activation of TLR-3 could recruit Myd88, Ticam-1 and Ticam-2 for signal transductions, consequently leading to enhanced inflammation [51]. The up-regulated gene expressions of α5 and TRPM7 were attributed to the released Mg^2+^ from the Ti-0.625 Mg alloy on day 1. Previous investigation revealed that exogenous Mg^2+^ could enhance the activation of α5β1 by promoting the ligand-binding potential of α5β1 and α5β1-fibronectin interactions [52, 53]. Ion channels are essential for the effect of biological membranes [54]. TRPM7 is known to be highly responsible for cellular magnesium homeostasis [55]. The deletion of TRPM7 gene in cells was reported to result in intracellular magnesium deficiency [56]. Recently, Yang et al. [57] further proved that the increase of the Mg^2+^ concentration in culture medium significantly increased the Mg^2+^ concentration in macrophages and increased the expression of Mg-related TRPM7. Hence, the up-regulation of TRPM7 gene expression is related to the increased import of Mg^2+^ into cells. On day 5, up-regulation in gene expressions of integrin (α5 and β1) signaling, while down-regulation in the gene expressions of TLR (TLR-3, TLR-4, Myd88, Ticam-1 and Ticam-2) and Mg-transport (TRPM6) signaling were noticed in Ti-0.625 Mg group. As aforementioned, the increased gene expressions of α5 and β1 could be owing to the Mg^2+^ release from the Ti-0.625 Mg alloy. The reduced gene expression of TRPM6 is associated with the accumulation of Mg^2+^ within cells. Moreover, the accumulated intracellular Mg^2+^ was reported to down-regulate TLR signaling and lead to inhibited the anti-inflammatory response [58]. The schematical pathways involved in the Ti-0.625 Mg alloy-mediated macrophage activation is shown in Fig. 11.

### The relationship between macrophage phenotypes and their osteogenic potential

The results indicate that both M1- and M2-lile macrophages could contribute to osteogenesis. This result is consistent with the result reported by Guo et al. that the Cu-Zn double-layer nanofiber membranes have dual temporal bidirectional immunomodulatory effects and have good anti-infection and osteogenic abilities [59]. According to the results presented in Fig. 8, the influence of environmental pH and Mg^2+^ on SaOS-2 cell maturation could be ruled out. The enhanced osteogenic potential of activated macrophages is attributed to the increased release of growth factors including BMP-2 and BMP-6 [7]. The results presented in Fig. 5 showed that both M1 and M2 macrophages activated secreted larger amounts of BMP-2 and BMP-6 than those in the Ti group. Consistent with our findings, Yu et al. investigated the osteogenic potential of M0, M1 and M2 macrophages and found that all the macrophage subtypes promoted osteogenesis [60]. Guihard et al. reported that the increased secretion of OSM by LPS-activated M1 macrophages was responsible for enhanced osteogenesis [19]. However, the gene expression of OSM in M1 macrophages activated by the Ti-0.625 Mg alloy was not up-regulated, indicating that the growth factor release profile of macrophages depends not only on their phenotype but also on the type of stimuli they receive.

The osteointegration between the Ti-0.625 Mg implant and bone tissue was investigated in our previous work [32]. The Ti-0.625 Mg implant was found to be more beneficial for osteointegration than the Ti implant. This study focused on the relationship between the inflammatory and osteogenesis in vivo. The results presented in Fig. 9 showed that the Ti-0.625 Mg implant resulted in a milder inflammatory response than the Ti implant, which was evidenced by the expressions of CD11c and CCL24. The positive relationship of the BMP-2 expression was also noticed on both day 7 and 28. The initial pro-inflammatory potential of the Ti-0.625 Mg implant compared with that of the control was not observed. This is probably because that the acute inflammatory response was already resolved on day 7 [61]. In general, the in vivo results were largely consistent with the in vitro results in the current work. The results presented in this work highlighted the osteogenic potential of M1 and M2 macrophages activated by the dynamic degradation of Mg.

## Conclusions

The Ti-0.625 Mg alloy fabricated by MA and subsequent SPS was employed as a material model to study the macrophage activation process in response to dynamic Mg degradation by taking Ti as the control. The results indicated that the Ti-0.625 Mg alloy sequentially activated M1 and M2 phenotypes in macrophages after being cultured for 1 and 5 days, respectively. Both M1 and M2 macrophages activated by Mg degradation in the Ti-0.625 Mg alloy promoted the maturation of SaOS-2 cells. In vivo results showed that the Ti-0.625 Mg alloy resulted in a milder inflammatory response than Ti. In addition, the expression of BMP-2 was found to be positively related. Together, the results highlighted the roles of Mg degradation in the Ti-0.625 Mg alloy on the sequential activation of macrophage phenotypes and the importance of facilitating the M1-to-M2 transition in macrophages for enhanced osteogenesis.

## Supplementary Information

**Additional File 1 .**

## Data Availability

All data are available on request.
