# Supplementary material for: Sequential activation of M1 and M2 phenotypes in macrophages by Mg degradation from Ti-Mg alloy for enhanced osteogenesis
Source: Biomater Res. 2022 Apr 28;26:17. doi: 10.1186/s40824-022-00262-w (PMC9052665; doi:10.1186/s40824-022-00262-w)
Supplement: Supplementary file 1 — Additional File 1 . [file 40824_2022_262_MOESM1_ESM.docx]

**[Supplementary Information](https://mc03.manuscriptcentral.com/scms?DOWNLOAD=TRUE&PARAMS=xik_JEm2KWTansn4bJaf2hTN9txZRAp6QpA9fg6eo7rKUcVtpHRFLYJLssb1ShPnXqbiQ3NyUWcwmduFeugnruXAwA1Cu94zGMz3Ppf8vxEejBRUa9Hi9oBZhsNSeXyKFyngdDSZubiX7zHrXJW1YyBagJrp7Jr29vYWDTyLT8swTJQMcSW)**

Sequential Activation of M1 and M2 Phenotype in Macrophages by Mg Degradation from Ti-Mg Alloy for Enhanced Osteogenesis

Luxin Liang ^a,b^, Deye Song ^b^, Kai Wu ^c^, Zhengxiao Ouyang ^b^, Qianli Huang ^a, d**^,

Guanghua Lei ^e^, Kun Zhou ^f^, Jian Xiao ^g, ***^, Hong Wu ^a, *^

^a^ State Key Laboratory of Powder Metallurgy, Central South University, Changsha 410083, P. R. China

^b^ Department of Orthopedics, The Second Xiangya Hospital, Central South University, Changsha 410011, P. R. China

^c^ Department of Rehabilitation, Xiangya Hospital, Central South University, Changsha 410008, P. R. China

*^d^ Foshan (Southern China) Institute for New Materials, Foshan 528200, P. R. China*

^e^ Department of Orthopedics, Xiangya Hospital, Central South University, Changsha, 410008, P. R. China

^f^ School of Mechanical and Aerospace Engineering, Nanyang Technological University, Singapore, 639798, Singapore

^g^ Department of Pharmacy, Xiangya Hospital, Central South University, Changsha, 410008, P. R. China

^h^ National Clinical Research Center for Geriatric Disorders, Xiangya Hospital, Central South University, Changsha 410008, P. R. China

E-mail: [hwucsu@csu.edu.cn](mailto:hwucsu@csu.edu.cn) (Hong Wu), [hql1990@163.com](mailto:hql1990@163.com) (Qianli Huang), [admanoas@163.com](mailto:admanoas@163.com) (Jian Xiao)


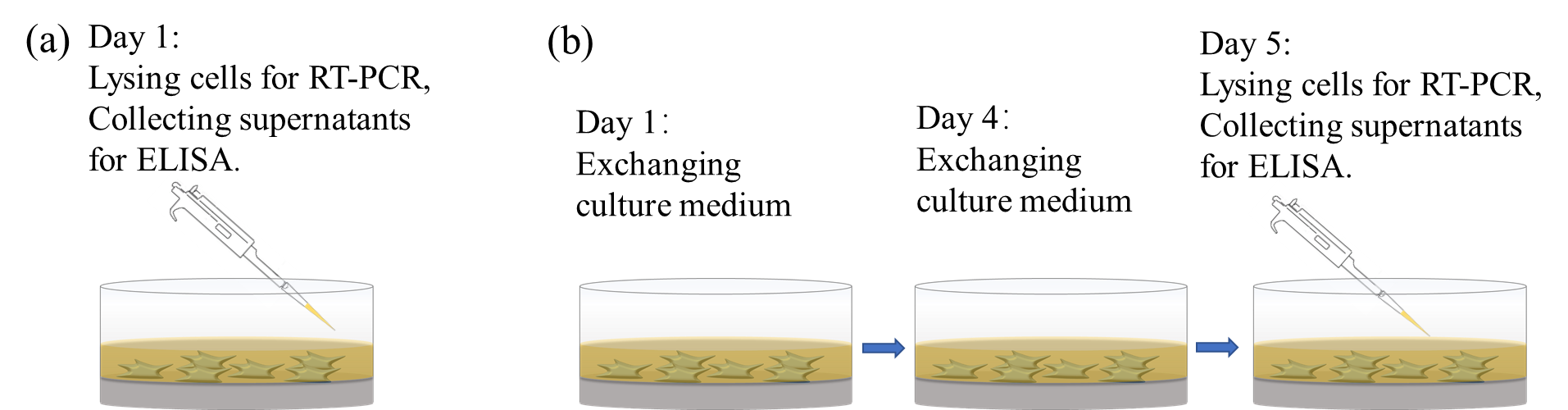


Fig. S1. The schematic showing the measured method of ELISA and RT-PCR for (a) day 1, and (b) day 5.

Table S1 The sequences of primers employed for macrophages

| Gene | Primer sequences |
| --- | --- |
| GAPDH | Forward: 5’-TCAGCAATGCCTCCTGCAC-3’ |
|  | Reverse: 5’-TCTGGGTGGCAGTGATGGC-3’ |
| CD86 | Forward: 5’-CTGCTCATCATTGTATGTCAC-3’ |
|  | Reverse: 5’-ACTGCCTTCACTCTGCATTTG-3’ |
| CD11c | Forward: 5’-ACTTCACGGCCTCTCTTCC-3’ |
|  | Reverse: 5’-CACCAGGGTCTTCAAGTCTG-3’ |
| iNOS | Forward: 5’-CAGAAGTGCAAAGTCTCAGACAT-3’ |
|  | Reverse: 5’-GTCATCTTGTATTGTTGGGCT-3’ |
| CCL24 | Forward: 5’-AGGCAGGGGTCATCTTCATCAC-3’ |
|  | Reverse: 5’-GGCTGGTTTTTCTTGGCATCC-3’ |
| IL-1β | Forward: 5’-TGGAGAGTGTGGATCCCAAG-3’ |
|  | Reverse: 5’-GGTGCTGATGTACCAGTTGG-3’ |
| TNF-α | Forward: 5’-CTGAACTTCGGGGTGATCGG-3’ |
|  | Reverse: 5’-GGCTTGTCACTCGAATTTTGAGA-3’ |
| IL-10 | Forward: 5’-TGCTATGCTGCCTGCTCTTA-3’ |
|  | Reverse: 5’-TGTTGTCCAGCTGGTCCTTT-3’ |
| BMP-2 | Forward: 5’-GCTCCACAAACGAGAAAAGC-3’ |
|  | Reverse: 5’-AGCAAGGGGAAAAGGACACT-3’ |
| BMP-6 | Forward: 5’-TGGCAGGACTGGATCATTGC-3’ |
|  | Reverse: 5’-ACCAAGGTCTGTACAATGGCG-3’ |
| OSM | Forward: 5’-ACGGTCCACTACAACACCAG-3’ |
|  | Reverse: 5’-CCATCGTCCCATTCCCTGAAG-3’ |
| α5 | Forward: 5’-CTTCTCCGTGGAGTTTTACCG-3’ |
|  | Reverse: 5’-GCTGTCAAATTGAATGGTGGTG-3’ |
| αM | Forward: 5’-CCATGACCTTCCAAGAGAATGC-3’ |
|  | Reverse: 5’-ACCGGCTTGTGCTGTAGTC-3’ |
| β1 | Forward: 5’-CGTGGTTGCCGGAATTGTTC-3’ |
|  | Reverse: 5’-ACCAGCTTTACGTCCATAGTTTG-3’ |
| β2 | Forward: 5’-CAGGAATGCACCAAGTACAAAGT-3’ |
|  | Reverse: 5’-GTCACAGCGCAAGGAGTCA-3’ |
| TLR-3 | Forward: 5’-CGGGATTGGTGAGTCTGAAG-3’ |
|  | Reverse: 5’-CGGAAAGGTGAAGGGGAG-3’ |
| TLR-4 | Forward: 5’-CACCTGATACTTGCTGGC-3’ |
|  | Reverse: 5’-TCCCCAGAGGATTGTCCTC-3’ |
| Myd88 | Forward: 5’-AGGTAAGCAGCAGAACCAGG-3’ |
|  | Reverse: 5’-TGTCCTAGGGGGTCATCAAGG-3’ |
| Ticam-1 | Forward: 5’-AGATGGTTCAGCTGGGTGTC-3’ |
|  | Reverse: 5’-TGGAGTCTCAAGAAGGGGTTC-3’ |
| Ticam-2 | Forward: 5’-CTTGGCGCTGCAAACCATC-3’ |
|  | Reverse: 5’-GCCTCTCAAATACAGACTCCCG-3’ |
| TRPM6 | Forward: 5’-TGGACTTCTTTGCTGTGAAT -3’ |
|  | Reverse: 5’-AAGAGGTAGACAGCTTGCA-3’ |
| TRPM7 | Forward: 5’-CTCATGGGAGGAACCTACAG-3’ |
|  | Reverse: 5’-CATCTTTGGTCTGTAGGGTTG-3’ |


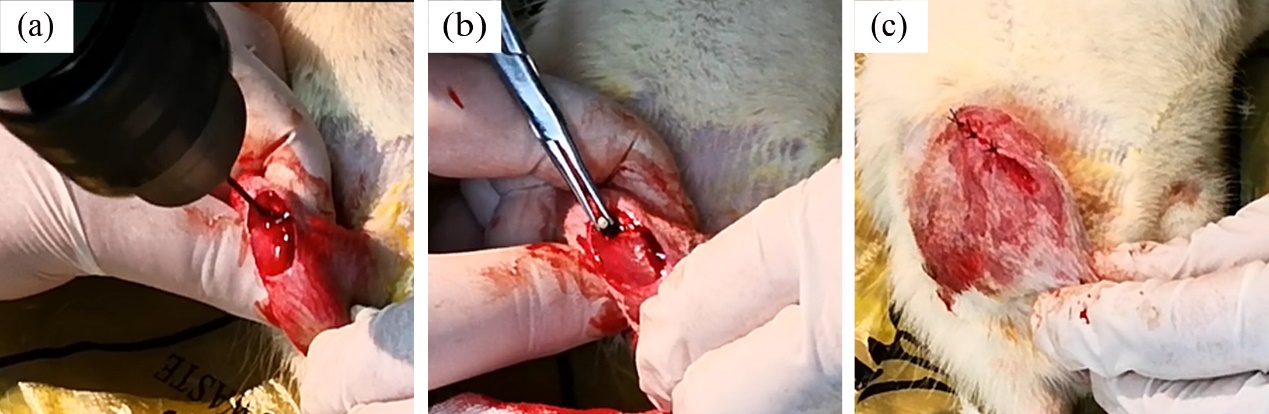


Figure. S2 Surgery procedures; (a) the defects of the femurs in longitudinal axis created by an electric drill, (b) implantation of Ti-Mg alloy in pro-dirlled defect and (c) sutured wound.
